# Supplementary material for: The Association of Malnutrition and Health-Related Factors among 474,467 Older Community-Dwellers: A Population-Based Data Mining Study in Guangzhou, China
Source: Nutrients. 2024 Apr 29;16(9):1338. doi: 10.3390/nu16091338 (PMC11085532; doi:10.3390/nu16091338)
Supplement: Supplementary file 1 [file nutrients-16-01338-s001.zip › nutrients-2933443-supplementary.pdf]

Supplement

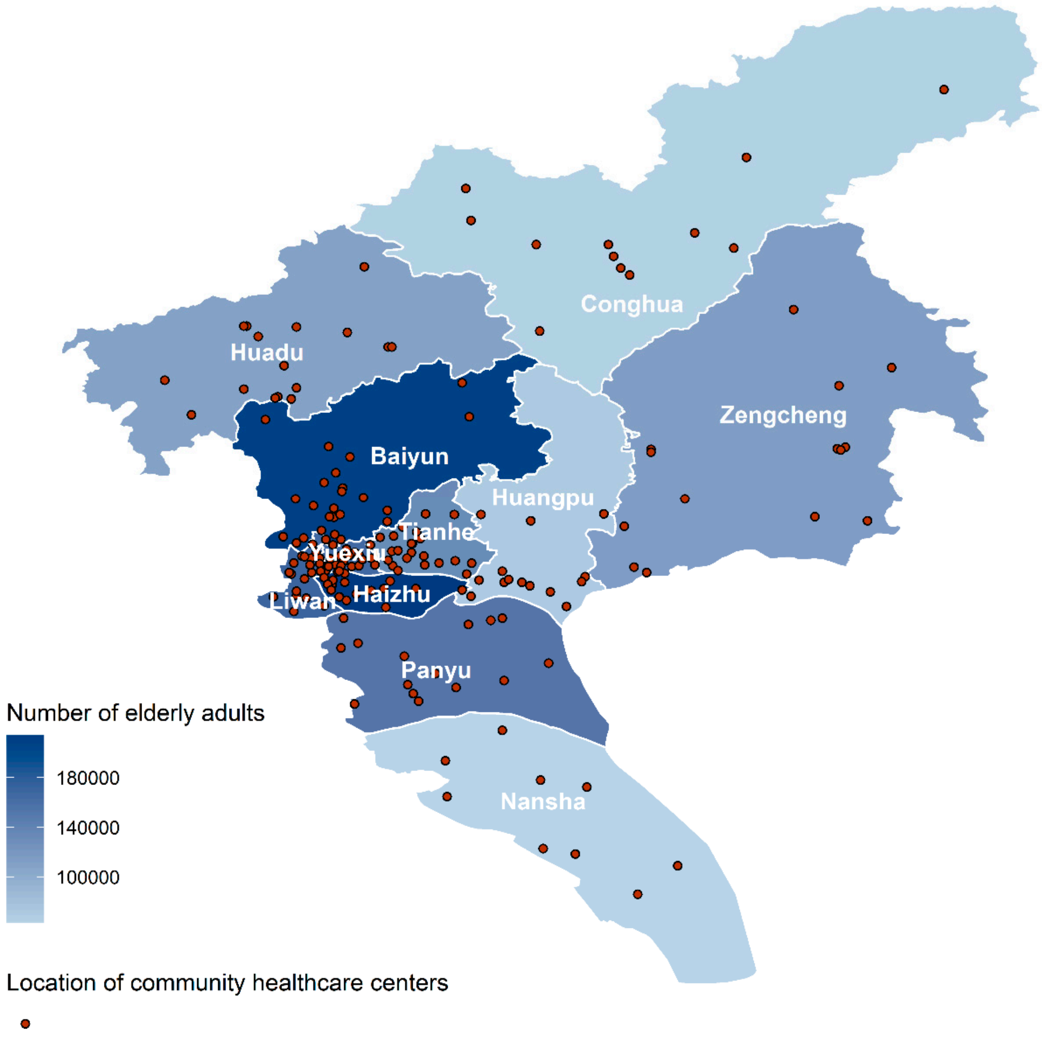

Figure S1. Distribution of the number of elderly adults by districts and location of community health centers in Guangzhou, China.

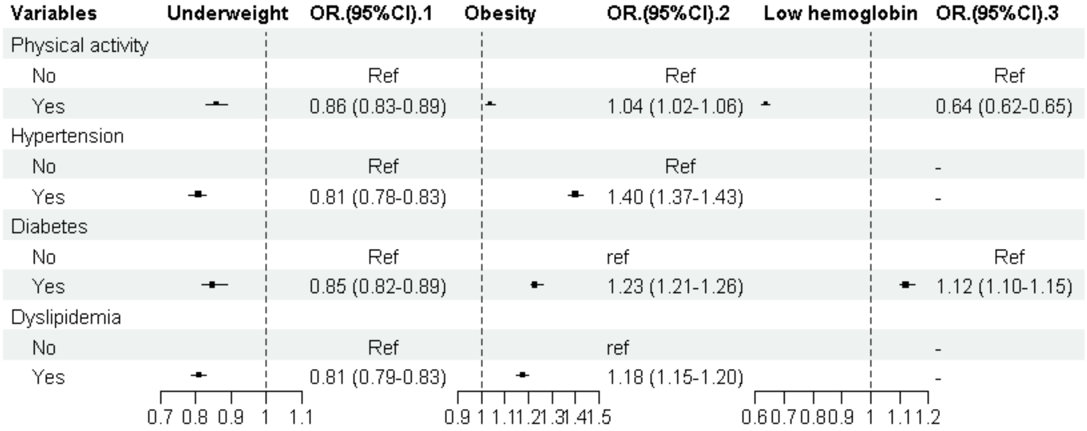

Figure S2. Association factors with three patterns of malnutrition explored by multivariate logistic regression with “dietary habits”removed*.

*: The model was adjusted for all socio-demographic characteristics including age, gender, census register, living areas, education level, marital status, and medical insurance.

OR-odd ratio, CI-confidence interval.

Table S1. Comparison of the study population and the seventh national population census of Guangzhou in 2020.

Variables	Study population, % (n =474,467)	Seventh national population census, % (n =1,460,333)[a]
**Age**		
65-69	40.33	39.44
70-74	28.49	24.58
75-79	15.51	14.74
80-84	9.42	11.30
≥85	6.25	9.94
**Gender**		
Male	41.21	46.16
Female	58.79	53.84

a: The categories are grouped according to the Seventh National Population Census of Guangzhou: National Bureau of Statistics of China, 2020.

Table S2. Results of stepwise analysis for underweight, overweight, and low hemoglobin*

Characteristics	Underweight OR (95%CI)	Overweight OR (95%CI)	Low hemoglobin OR (95%CI)
**Age** (ref:65-69)			
70-74	0.99 (0.96-1.03)	0.97 (0.95-1.00)	1.03 (1.00-1.06)
75-79	1.07 (1.03-1.12)	0.96 (0.93-0.99)	1.08 (1.04-1.11)
80-84	1.04 (0.99-1.09)	0.91 (0.88-0.94)	1.19 (1.15-1.24)
≥85	1.17 (1.10-1.24)	0.84 (0.80-0.87)	1.41 (1.35-1.47)
**Gender** (ref: Male)			
Female	1.02 (0.99-1.05)	1.08 (1.06-1.10)	0.41 (0.40-0.41)
**Census register** (ref: Guangzhou)			
Non-Guangzhou	0.86 (0.81-0.91)	1.01 (0.98-1.05)	0.87 (0.84-0.91)
**Living area** (ref: Urban)			
Rural	1.10 (1.07-1.14)	1.08 (1.06-1.11)	1.18 (1.15-1.21)
**Education** (ref: No school)			
Primary	0.87 (0.83-0.91)	1.08 (1.04-1.12)	0.79 (0.76-0.81)
Secondary	0.78 (0.74-0.82)	0.90 (0.87-0.94)	0.55 (0.53-0.58)
College	0.82 (0.77-0.86)	1.11 (1.07-1.15)	0.84 (0.81-0.87)
**Marital status** (ref: Single)			
Married	0.81 (0.78-0.85)	1.05 (1.02-1.09)	0.56 (0.55-0.58)
**Medical insurance** (ref: Uninsured)			
Insured	1.21 (1.10-1.31)	1.06 (1.01-1.12)	1.24 (1.17-1.33)
**Current smoking** (ref: No)			
Yes	1.44 (1.39-1.51)	0.55 (0.53-0.57)	0.58 (0.55-0.60)
**Alcohol consumption** (ref: No)			
Yes	0.79 (0.75-0.84)	1.21 (1.17-1.26)	0.55 (0.53-0.58)
**Physical activity** (ref: No)			
Yes	0.87 (0.84-0.90)	1.03 (1.01-1.05)	0.66 (0.64-0.67)
**Dietary habits** (ref: Balanced)			
Meat or fish diet	0.91 (0.73-1.13)	1.94 (1.74-2.17)	0.78 (0.66-0.92)
Vegetarian diet	1.01 (0.91-1.13)	1.00 (0.93-1.07)	0.91 (0.84-0.99)
**Hypertension** (ref: No)			
Yes	0.81 (0.79-0.83)	1.39 (1.36-1.42)	0.96 (0.94-0.99)
**Diabetes** (ref: No)			
Yes	0.85 (0.82-0.89)	1.23 (1.21-1.26)	1.13 (1.10-1.16)
**Dyslipidemia** (ref: No)			
Yes	0.81 (0.79-0.84)	1.17 (1.15-1.20)	0.84 (0.82-0.86)

*: This Table also shows the OR and 95% CI for socio-demographic characteristics while Figure 2 in the manuscript does not because we concentrate on health-related factors in this study.
